# Brain elastography in aging relates to fluid/solid trendlines

**DOI:** 10.1088/1361-6560/ad4446

**Published:** 2024-05-29

**Authors:** Kevin J Parker, Irteza Enan Kabir, Marvin M Doyley, Abrar Faiyaz, Md Nasir Uddin, Gilmer Flores, Giovanni Schifitto

**Affiliations:** 1 Department of Electrical and Computer Engineering, University of Rochester, 724 Computer Studies Building, Box 270231, Rochester, NY 14627, United States of America; 2 Department of Biomedical Engineering, University of Rochester, 204 Goergen Hall, Box 270168, Rochester, NY 14627, United States of America; 3 Department of Imaging Sciences, University of Rochester Medical Center, 601 Elmwood Ave, Box 648, Rochester, NY 14642, United States of America; 4 Department of Neurology, University of Rochester Medical Center, 601 Elmwood Ave, Box 673, Rochester, NY 14642, United States of America

**Keywords:** brain, viscoelastic models, biphasic models, MRI, elastography, aging

## Abstract

The relatively new tools of brain elastography have established a general trendline for healthy, aging adult humans, whereby the brain’s viscoelastic properties ‘soften’ over many decades. Earlier studies of the aging brain have demonstrated a wide spectrum of changes in morphology and composition towards the later decades of lifespan. This leads to a major question of causal mechanisms: of the many changes documented in structure and composition of the aging brain, which ones drive the long term trendline for viscoelastic properties of grey matter and white matter? The issue is important for illuminating which factors brain elastography is sensitive to, defining its unique role for study of the brain and clinical diagnoses of neurological disease and injury. We address these issues by examining trendlines in aging from our elastography data, also utilizing data from an earlier landmark study of brain composition, and from a biophysics model that captures the multiscale biphasic (fluid/solid) structure of the brain. Taken together, these imply that long term changes in extracellular water in the glymphatic system of the brain along with a decline in the extracellular matrix have a profound effect on the measured viscoelastic properties. Specifically, the trendlines indicate that water tends to replace solid fraction as a function of age, then grey matter stiffness decreases inversely as water fraction squared, whereas white matter stiffness declines inversely as water fraction to the 2/3 power, a behavior consistent with the cylindrical shape of the axons. These unique behaviors point to elastography of the brain as an important macroscopic measure of underlying microscopic structural change, with direct implications for clinical studies of aging, disease, and injury.

## 1. Introduction

Decades of research on the aging human brain have revealed a wide range of information about long term changes in morphology, anatomy, and composition. A more recent addition comes from the application of elastography, where magnetic resonance imaging (MRI) and other imaging platforms are adapted to measure the stiffness and viscoelastic properties of tissue, *in vivo* (Ormachea and Parker 2020). While elastography research has established some basic trends versus age for healthy subjects, we still lack a detailed understanding of the dominant mechanisms that cause long-term changes in brain viscoelasticity in the healthy human adult. This study examines some of the most prominent changes known to be present in the aging brain, from 20’s to 80’s+ years (adult aging), and then seeks to derive a quantitative link to the elastography trends reported herein and previously.

**Figure 1.**
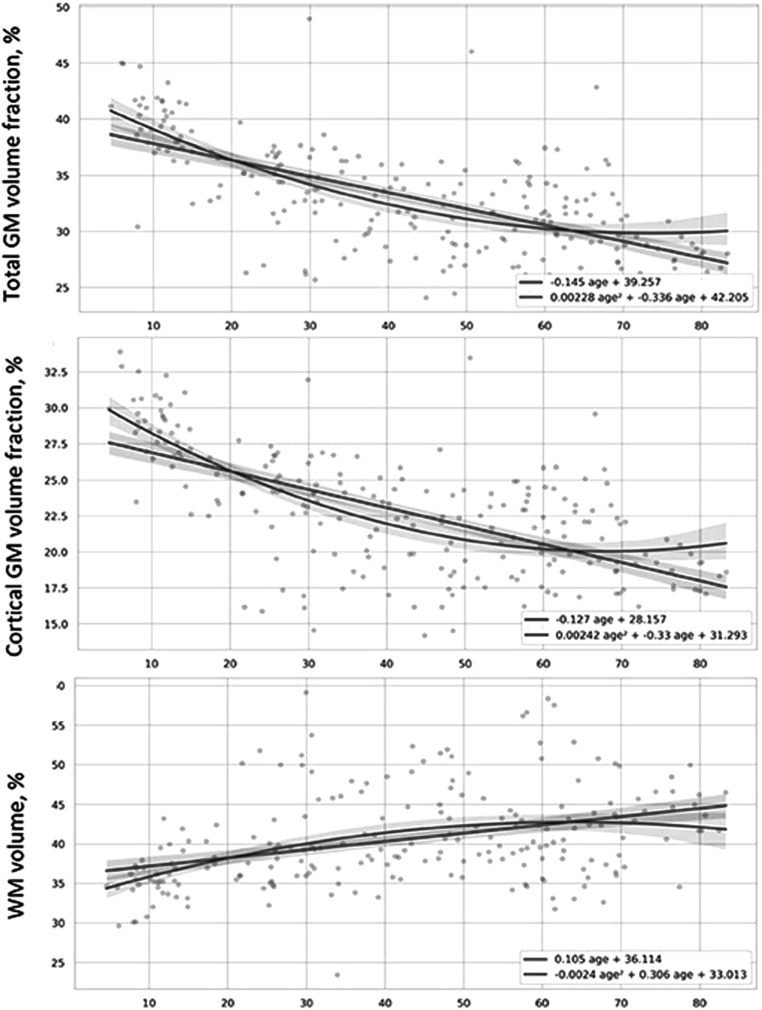
Distribution of results of voxel-based brain morphometry (VBM) over age. (Top) total grey matter volume fraction. (Middle) cortical grey matter volume fraction. (Bottom) white matter volume. Red lines show linear, and green lines second order, fits to the data, respectively (Statsenko *et al* 2021).

It has been widely recognized that at a gross level of anatomical structure and size, a highly visible (on MRI) effect of aging is the onset and progression of the grey matter atrophy. This is associated with an increase in the proportion of total cerebrospinal fluid (CSF) and intraventricular CSF (iCSF) (Giorgio *et al* 2010, Statsenko *et al* 2021). Figure 1 shows a sharp decrease in grey matter volume fraction contrasted with the slightly increasing trend for the white matter volume fraction, as a function of age in humans.

It can also be seen from the literature that many physical, chemical, anatomical, and MRI-related parameters and measures have been studied in the brain as a function of age, for instance potassium, calcium, and iron (Hallgren and Sourander 1958, Hebbrecht *et al* 1999), and myelin content (Schmierer *et al* 2004, 2007, Billiet *et al* 2015, Bouhrara *et al* 2020). In addition, there is the overall state of inflammation and dysregulation of the glymphatic system of the brain as a function of age that is still under active investigation (Jessen *et al* 2015, Benveniste *et al* 2019, Dai *et al* 2023). Thus, the complex, multiscale, long-term changes in the aging brain contain numerous possible influences on the stiffness of the brain as a function of age, as measured by elastography techniques.

**Figure 2.**
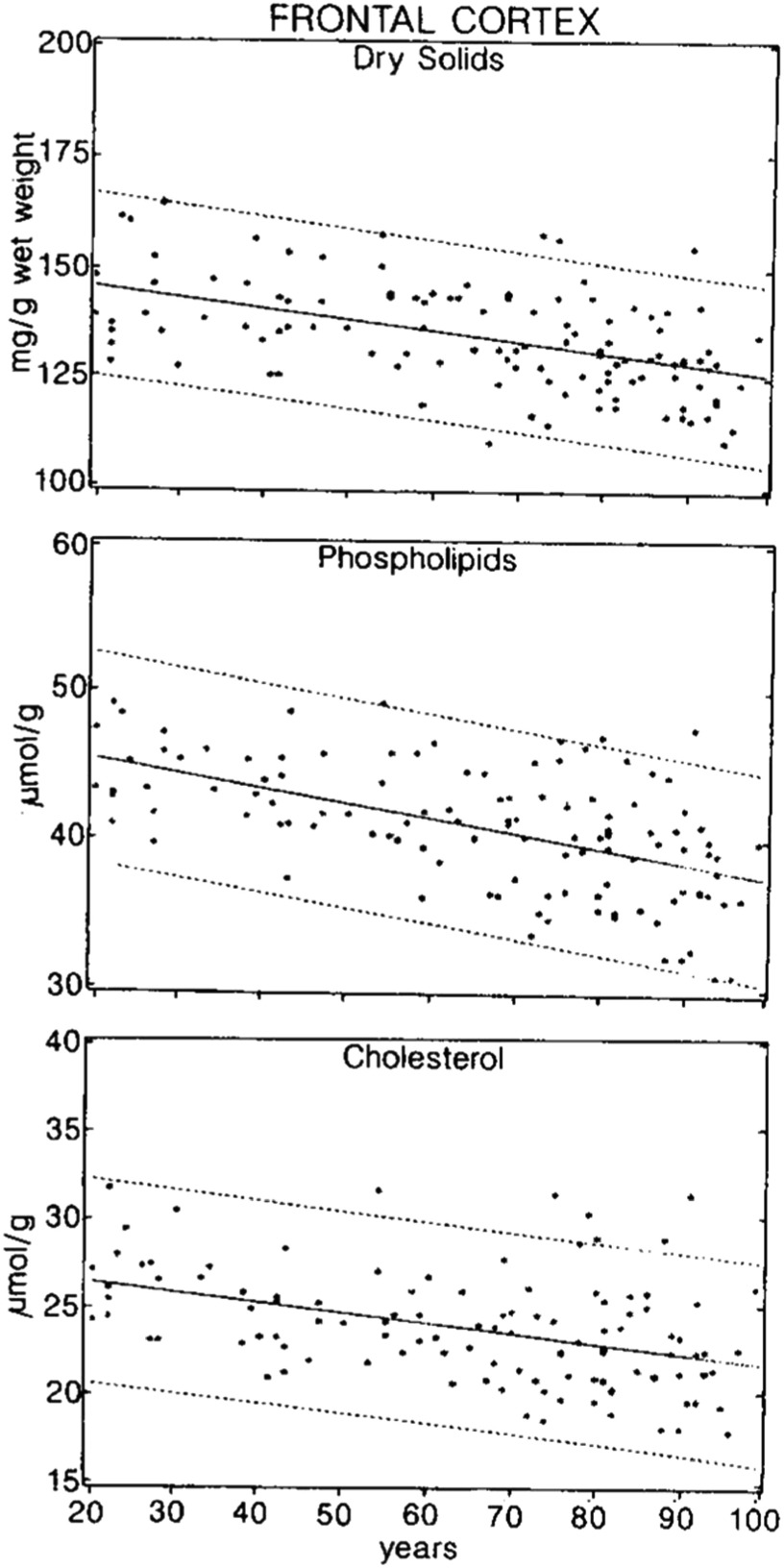
Concentration of dry solids, phospholipids, and cholesterol in frontal cortices of subjects, age 20–100 years (Svennerholm *et al* 1994).

Turning more specifically to elastography and the viscoelastic properties of the brain, a key component in all models of rheology and many MRI measures is the percent of water and its distribution within a supporting matrix. A landmark study of the dry/wet weight percent along with the fractions of important cellular and extracellular components, as a function of age, was conducted by Svennerholm *et al* (1994) for a carefully selected group of individuals without pre-existing neuropsychiatric conditions. The postmortem samples demonstrated major decreases (on the order of 20%–25%) in the fraction of total dry solids, including phospholipids and cholesterol, in grey matter as a function of age from 20 to 100 years, as reproduced in figure 2. Similar reductions were found in white matter, but with a delay as to the onset of the decline, initiating later into middle age and then accelerating with old age.

In parallel with these anatomical and chemical measures of the aging brain, the newer *in vivo* measures of elastography using shear waves generally show a trend towards decreasing brain stiffness as a function of adult age (Hiscox *et al* 2018, Arani *et al* 2021, Hiscox *et al* 2021). The key question emerging from the above observations is: which particular mechanisms contribute to the decreasing viscoelastic trend lines as the human brain ages into its 80s and 90s? The answer is important beyond empirical correlations, because the causal link of elastography measures to specific mechanisms will create an understanding of what elastography can uniquely identify, beyond that available with other MRI-derived parameters for assessment of aging, pathologies, and response to treatments.

In order to winnow down the large possible set of contributors to the decreasing brain stiffness versus age, and to identify a more focused set of dominant mechanisms which plausibly set the trendline, we utilize a multiscale biphasic model of fluid channels in an otherwise elastic parenchymal matrix, generically called the ‘microchannel flow model’ (MFM). Within the framework created by the model, we examine and attempt to model key changes in the elastic nature of the brain as a function of age using shear wave elastography techniques presented in Kabir *et al* (2023), Kabir (2023).

The following sections first review the mathematical methods applied to the biphasic, multiscale rheological model of the brain, then the experimental methods and results from MRI including diffusion tensor imaging (DTI) and neurite orientation dispersion and density imaging (NODDI), and finally the plausible match between theory and experimental observations. The key role of extracellular free water, its distribution and quantification, along with the gradual change in composition of the brain, are seen to be influential in creating a decreasing overall trendline versus age for grey matter stiffness, but with some modifications noted for white matter. Numerous studies have leveraged diffusion MRI (DTI and NODDI) to explore the impact of aging on brain microstructure (Pfefferbaum and Sullivan 2003, Davis *et al* 2009, Sexton *et al* 2014, Nazeri *et al* 2015, Cox *et al* 2016, Merluzzi *et al* 2016, Miller *et al* 2016, Guerreri *et al* 2019, Slater *et al* 2019). While a consistent age-related increase in free water has been noted, earlier research has revealed some discrepancies concerning changes in neurite density index (NDI) and orientation dispersion index (ODI), likely due to variations in the age groups studied (Billiet *et al* 2015, Kodiweera *et al* 2016, Merluzzi *et al* 2016). In an exceptionally extensive dataset, a confirmed decline in NDI was observed from middle to old age, while ODI generally exhibited a nonlinear increase until around 60 years, followed by a decrease (Cox *et al* 2016). Previous studies (Cox *et al* 2016, Slater *et al* 2019) consistently highlight mean diffusivity (MD) as the most sensitive among diffusion MRI parameters (fractional anisotropy (FA), MD, NDI, ODI, and extracellular free water (FW)) to aging effects. Furthermore, other studies have incorporated myelin-sensitive quantitative MRI data, emphasizing the necessity of integrating complementary techniques to fully understand the temporal and spatial heterogeneity across tracts (Billiet *et al* 2015, Slater *et al* 2019, Faizy *et al* 2020).

## 2. Theory

We derived the stress–strain behavior of the brain as a two-component model with a larger scale network representing the vascular and perivascular branching structures, down to the level of capillaries, and then including an additional version representing the smaller scale fluid channels within the interstitial spaces, in particular the glymphatic system. These parameters are conditioned by anatomical measures, including a key power law parameter *a* that captures the multiscale fractal nature of the fluid channels within the soft tissue parenchyma, discussed in more detail in Ge *et al* (2022, 2023). The complex Young’s modulus as a function of frequency is then derived by Laplace transform theory as the sum of components. However, a simplified form for the stiffness versus frequency is possible in the typical range of elastography frequencies (40–200 Hz for adult human brains) and so our working approximation for the complex Young’s modulus $E$ is:

\begin{eqnarray*}E\left(\omega \right)={A}_{1}{\left(-I\omega \right)}^{a}+{A}_{2},\end{eqnarray*}

where $\omega $ is the radial frequency of the shear waves employed in elastography, $I$ is the imaginary unit, $a> 0$ is the power law exponent linked to the distribution of fluid channel sizes, and ${A}_{1}$ and ${A}_{2}$ represent the relative contributions from the vascular and glymphatic portions of the fluid channels responding to stress and strain, respectively, within the parenchymal matrix. The basic model can similarly be written for shear modulus $G\left(\omega \right),$ under the assumption that the elastic matrix or tissue parenchyma is nearly incompressible such that $G\left(\omega \right)=E\left(\omega \right)/3$ (Parker 2017b). The shear wave speed (SWS) as a function of frequency (the phase velocity and dispersion) can also be calculated from this quantity. In this equation, the ${A}_{2}$ term is an asymptotic approximation to the glymphatic terms that are characterized by very long time constants valid over the typical range of frequencies used in elastography. This is a result of the time constants within the glymphatic system being so long as to result in a nearly constant term over the typical range of brain elastography experiments above 10 Hz shear excitations.

Next, we consider dilation or constriction of the fluid channels within either of the two compartments. It can be shown that this change can be treated simply with scale factors shifting both the magnitude and the time constants associated with the network of fluid channels.

If all the fluid-filled spaces within the glymphatic system (and correspondingly the water fraction) are increased or decreased by a factor of ${r}_{2}=\chi r$ where $\chi > 1$ represents dilation and $\chi < 1$ represents constriction of nominal radius or axis $r,$ then we can determine in theory how this affects the complex Young’s modulus and shear modulus $G.$ Furthermore, if the elastic properties of the cellular structures change, without any alteration of vessel diameters we can account for that change as well. Electro-chemical influences on various cells, axons, dendritic spines, cell membranes, and actin filaments have been reviewed by Tyler (2012) and Barnes *et al* (2017). Functional stimuli may incite regional electro-chemical changes (Patz *et al* 2016). Thus, our model recognizes that the intracellular water content, bound water, and components including myelin and proteoglycans (Svennerholm *et al* 1994) will not be a constant across the different decades of life, so the model’s baseline parenchymal stiffness will itself have a trend as a function of age.

It is convenient to assume a baseline case of shear modulus $G$ at a reference age or condition, then a modified shear modulus ${G}_{2}={\chi }_{E}G$ is observed in the elastic matrix, captured by the fractional factor of ${\chi }_{E},$ and this modification can be can be derived through the transformation rules (Parker 2017a).

Now summarizing and combining these influences for the brain model (Ge *et al* 2023), the leading terms in the equations for the complex modulus can be written approximately as:

\begin{eqnarray*}{G}_{2}=\displaystyle \frac{{G}_{0}{\chi }_{E}^{\left(1-a\right)}}{{\chi }^{\left(1.5a\right)}},\end{eqnarray*}

where ${G}_{2}$ is the altered modulus as $\chi $ and ${\chi }_{E}$ are varied around a reference point of 1 corresponding to a reference modulus of ${G}_{0}.$ We next hypothesize that ${\chi }_{E}$ can be approximated as inversely proportional to the fractional change in water volume fraction $\unicode{x02206}W,$ which will change over time proportional to ${\chi }^{3}$ in our model (dilated radii contain increased water volumes, and volume is proportional to radius to the cube power for spherical shapes). A stronger relationship would be to have the parenchymal stiffness vary as inversely proportional to ${\unicode{x02206}W}^{2},$ where ${\mathrm{\Delta }}W$ is the water volume fraction or ${\left({\chi }^{3}\right)}^{2}.$ This square term has some support from measured dependence of biomaterials as a function of water/solid content (Nguyen *et al* 2014), depending on the exact formulation used. Assuming the square dependence and combining the terms yields an approximate expression for long term changes of the complex modulus ${G}_{2}$ in the brain from some nominal value ${G}_{0},\,$ where ${G}_{2}$ is given as:

\begin{eqnarray*}{G}_{2}=\displaystyle \frac{{G}_{0}}{{\chi }^{\left(1.5a+\left(1-a\right)6\right)}}=\displaystyle \frac{{G}_{0}}{{\chi }^{\left(6-4.5a\right)}},\end{eqnarray*}

and, for example, if $a=0.05,$ then the denominator term is ${\chi }^{5.75},$ a very strong dependence of the modulus with variations in fluid content. It must be understood that equation (3) represents a first order approximation of small changes in $\chi $ and water volume around a reference state in an elastic matrix that contains a multiscale or fractal branching network of fluids.

## 3. Methods

### 3.1. Subject demographics

MR images were obtained from 28 healthy subjects with age ranging from 22 to 79 years, who had no neurological disorders. The study was reviewed and approved by the Research Subjects Review Board (RSRB #67634) at the University of Rochester, and informed consent was obtained from the study participants. A summary of the patient demographics is given in table 1.

**Table 1.** Subject demographics.

Age range (years)	Mean age (years ± SD)	Number in age range	Number, gender = male	Number, gender = female	Number, race = white	Number, race = non-white
20–29	23.50 ± 1.50	2	2	0	2	0
30–39	31.00 ± 1.00	2	2	0	0	2
40–49	46.75 ± 2.86	4	3	1	3	1
50–59	55.40 ± 2.73	10	8	2	7	3
60–69	67.50 ± 1.89	6	3	3	5	1
70–79	72.25 ± 2.49	4	2	2	4	0
Total		28	20	8	21	7

### 3.2. Image acquisition

All imaging was conducted on a research-dedicated 3T whole-body Siemens Prisma scanner (Erlangen, Germany), equipped with a 64-channel receive-only head coil and body coil transmission, and high-performance gradients of max strength 80 mT m^−1^ and slew rate of 200 mT m^−1^ s^−1^.

#### 3.2.1. Anatomical imaging

T1-weighted images were collected using a 3D MPRAGE sequence with the following scan parameters: inversion time (TI) = 962 ms, repetition time/echo time (TR/TE) = 1840 ms/2.34 ms, field of view (FOV) = 256 × 256 mm^2^, number of slices = 176, and resolution = 1 × 1 × 1 mm^3^.

#### 3.2.2. MR elastography

The shear wave elastography was implemented on the MRI scanner. The shear wave excitations at 50 Hz were applied by a pair of air pads located under the subject’s head and activated by an air cylinder providing oscillating pressure. The waveform generator was triggered three seconds prior to the start of the imaging to ensure steady state shear wave propagation.

The tissue displacements were measured using a single-shot spin-echo planar imaging (SE-EPI) sequence (Hirsch *et al* 2014) with the following scan parameters: TR/ TE = 5100 ms/76 ms, number of slices = 45, FOV = 200 × 200 mm^2^, and resolution = 2 × 2 × 2 mm^3^. Eight phase offsets were taken, and a motion encoding gradient (MEG) amplitude of 70 mT m^−1^ was employed for the acquisitions.

#### 3.2.3. Diffusion MRI acquisition

Diffusion weighted images (DWI) were collected using a 2D single-shot SE-EPI pulse sequence via simultaneous multi-slice (SMS) acquisition technique with the following parameters: TR/TE = 4300 ms/69.0 ms, b = 1000, 2000 s mm^−2^, 64 directions/shell, GRAPPA = 2, multiband factor = 3, FOV = 256 × 256 mm^2^, number of slices = 96, resolution = 1.5 × 1.5 × 1.5 mm^3^. Seven T2-weighted reference images were acquired with a b-value = 0 s mm^−2^ to normalize DWIs with b > 0 s mm^−2^. Images with opposite polarity (i.e. AP and PA directions) were acquired to rectify DTI distortion.

#### 3.2.4. Myelin water imaging (MWI) acquisition

Whole brain MWI were performed using a 3D gradient and spin echo (GRASE) sequence with TR = 1000 ms, first TE = 10 ms, number of echoes = 32, echo spacing = 10 ms, number of slices = 24, slice thickness = 5 mm, and resolution = 1.5 × 1.5 × 5 mm^3^.

### 3.3. Image processing

Image pre-processing and analyzes were performed using FMRIB’s Software Library (FSL) (Jenkinson *et al* 2012), Advanced Normalization Tools (ANTs) (Avants *et al* 2009, 2011), MATLAB (version 2018b, The MathWorks, Inc., Natick, Massachusetts, United States) and Python (version 3.7.4). All raw images were checked for any severe artifacts such as gross geometric distortion, bulk motion, or signal dropout.

#### 3.3.1. MR elastography

Two independent techniques were employed to reconstruct local SWS and the reported $G$ is taken as the average of the two independent estimates: first, the multi-frequency dual elasto-visco (MDEV) reconstruction method (Papazoglou *et al* 2012, Hirsch *et al* 2014, Barnhill *et al* 2018, Meyer *et al* 2022), and secondly the reverberant shear wave autocorrelation technique (Parker *et al* 2017, Ormachea *et al* 2018, Ormachea and Zvietcovich 2021). The 3D-MDEV reconstruction method is a well-established direct inversion approach which can be applied to single or multiple frequency excitations. This method utilizes low-pass filters to mitigate noise interference (Papazoglou *et al* 2012, Hirsch *et al* 2014, Barnhill *et al* 2018, Meyer *et al* 2022). The reverberant shear wave elastography (RSWE) method is a relatively new technique which has been adapted to MRE with estimators based on the magnitude and phase of any component of an assumed 3D distribution of shear waves (Kabir *et al* 2023). Since these two techniques employ different approaches to the estimation of SWS (inverse Helmholtz for MDEV versus autocorrelation for RSWE), the combination is proposed as a means of conditioning the results against noise and artifacts.

#### 3.3.2. DTI/NODDI

The DWIs were corrected for eddy current-induced distortion, inter-volume subject motion, and susceptibility-induced distortion using ‘topup’ and ‘eddy’ tools in FSL (Andersson *et al* 2003, Andersson and Sotiropoulos 2016). DTI metrics such as FA and MD were computed by using DTIFIT in FSL (Smith *et al* 2004). FA indicates the degree of anisotropy or directionality of water diffusion in the tissues while MD is a measure of average water diffusion of water molecules in all directions.

A NODDI microstructural model was fitted using the NODDI toolbox (https://www.nitrc.org/projects/noddi_toolbox/) running in MATLAB which yields maps of NDI, ODI, and FW (Faiyaz *et al* 2022). NDI reflects neurite density, encompassing both axons and dendrites, while ODI represents the variability in neurite orientation. The FW index measures the relative fraction of freely diffusing water in the extracellular space, providing insights into neuroaxonal damage and its impact on tissue diffusion characteristics.

#### 3.3.3. MWI

Voxel-wise myelin water fraction (MWF) maps were computed using a regularized, non-negative least squares algorithm, while compensating for stimulated echoes, as reported previously (Prasloski *et al* 2012). In this way, MWF was calculated as the ratio of the T2 signal from 10 to 40 ms (i.e. myelin water) divided by the total T2 signal from 10 to 200 ms (i.e. myelin water plus intra/extracellular water). T2w GRASE images at 12th echo in native space were then co-registered (affine) with each participant’s bias-field corrected T1w image and then the brain masks were applied. The same brain masks were applied to MWF. Spatial normalization of the MWF map was performed using FSL.

### 3.4. Region of interest (ROI) analysis

FMRIB’s Automated Segmentation Tool (FAST) (Zhang *et al* 2001) was utilized to segment the brain regions, and registration employed ANTS (Avants *et al* 2011). Prior to segmentation, non-brain tissue was eliminated, and bias correction was performed using FSL’s brain extraction tool (BET) (Smith 2002). FSL’s brain anatomy pipeline, ‘fsl_anat,’ was employed to isolate brain tissues. The segmented regions included global grey and white matter, along with several subregions within subcortical grey matter, and white matter tract regions utilized for other studies. In addition, the Harvard–Oxford (subcortical, and cortical) and JHU-ICBM (white matter) atlases were used to calculate regional averages in standard space (1 mm) in pre-defined ROIs. MR metrics were extracted from global grey and white matter using corresponding masks. To mitigate partial volume effects caused by CSF in NODDI metrics such as NDI, ODI, and FW, we exclusively utilized the average values of all ROIs from the Harvard–Oxford cortical and subcortical GM atlas for assessing global grey matter. Similarly, we employed the average values of all ROIs from the JHU-ICBM atlas for analyzing global white matter.

### 3.5. Statistical analysis

The primary statistical approaches used in this study were the Pearson correlation and least squares error linear regression to assess the trends with age-related changes of shear modulus and diffusion MRI metrics (i.e. FA, MD, FW, NDI, ODI), as well as the degree of correlation between MRI metrics and shear modulus with aging as a parametric (implicit or hidden) parameter.

## 4. Results

The measured shear modulus and other MRI parameters are considered in three stages in the following sections. First, we examine the trends as a function of subjects’ age. Next, to assess deeper interrelationships, we plot the correlations between shear modulus and the other MRI measures. Finally, the trends for shear modulus are examined as a function of total dry/wet weight ratios as determined from the extensive studies of Svennerholm *et al* (1994).

### 4.1. Primary measures versus age and trendlines

**Figure 3.**
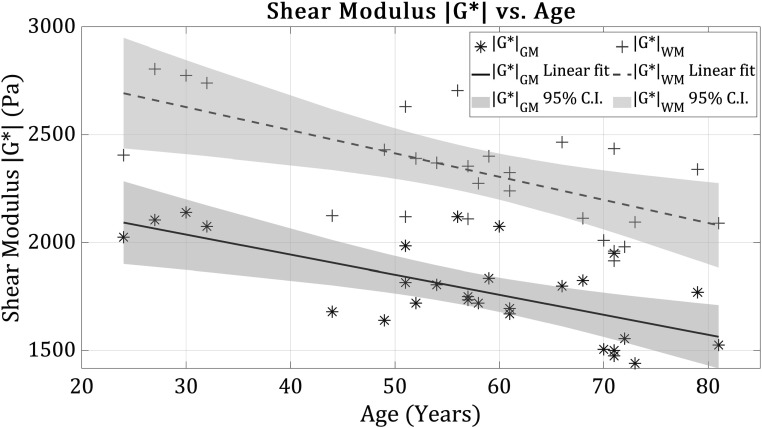
Shear modulus $\left|{G}^{* }\right|$ as a function of subjects’ age, reverberant + MDEV, for grey (GM, in blue) and white (WM, in red) matter. The overall trend is towards a softening of the brain in old age.

Figure 3 provides the shear modulus for grey and white matter as a function of age. Consistent with other studies from the literature (Arani *et al* 2015, Hiscox *et al* 2021), the white matter exhibits higher stiffness than grey matter, and the general trend lines indicate decreasing stiffness as a function of age across the adult lifespan.

Applying a linear fit to the data, the equations for $G$ as a function of age are given by:

\begin{eqnarray*}\begin{array}{rcl}{G}_{{\mathrm{grey}}}\left({\mathrm{age}}\right) &amp; = &amp; \left(-9.3\times {\mathrm{age}}\right)+2320\,{\mathrm{Pa}}\\ {G}_{{\mathrm{white}}}\left({\mathrm{age}}\right) &amp; = &amp; \left(-10.7\times {\mathrm{age}}\right)+2950\,{\mathrm{Pa}}\end{array}\end{eqnarray*}

Other parameters as a function of age are plotted in the appendix. However, we wish to focus on the most influential factors that strongly correlate with $G,$ that is with $r> 0.5$ or significant p-values < 0.05. Tables 2 and 3 list the correlations and normalized ranges of each of the measured parameters, and in bold indicate the few that lie above that level.

**Table 2.** Pearson’s correlation coefficients ($R$), *p*-values, and linear fit equations (grey matter).

*x*	*y*	$R$	*p*-value	Linear fit equation
	$\left|{G}^{* }\right|$	**−0.67**	**0.0001**	${\boldsymbol{y}}=-{\bf{9.29}}{\boldsymbol{x}}+{\bf{2.32}}{\times {\bf{10}}}^{{\bf{3}}}$
	ODI	−0.083	0.67	$y=-0.000137{x}+\,0.446$
Age	NDI	**0.59**	**0.00083**	${\boldsymbol{y}}={\bf{0.00118}}{\boldsymbol{x}}+{\bf{0.487}}$
	FA	−0.17	0.4	$y=-0.000135{x}+\,0.215$
	FW	**0.58**	**0.0018**	${\boldsymbol{y}}={\bf{0.00233}}{\boldsymbol{x}}+{\bf{0.273}}$
	MD	**0.48**	**0.022**	${\boldsymbol{y}}={\bf{1.15}}{\times {\bf{10}}}^{-{\bf{6}}}{\boldsymbol{x}}+{\bf{0.000718}}$

	ODI	0.096	0.63	$y=1.14{\times 10}^{-5}{x}+\,0.418$
	NDI	**−0.64**	**0.00024**	${\boldsymbol{y}}=-{\bf{9.16}}{\times {\bf{10}}}^{-{\bf{5}}}{\boldsymbol{x}}+{\bf{0.718}}$
$\left|{{\boldsymbol{G}}}^{* }\right|$	FA	**0.50**	**0.0096**	${\boldsymbol{y}}={\bf{2.74}}{\times {\bf{10}}}^{-{\bf{5}}}{\boldsymbol{x}}+{\bf{0.159}}$
	FW	**−0.75**	${\bf{1.2}}\times {{\bf{10}}}^{-{\bf{5}}}$	${\boldsymbol{y}}=-{\bf{0.000208}}{\boldsymbol{x}}+{\bf{0.777}}$
	MD	**−0.70**	${\bf{5.8}}\times {{\bf{10}}}^{-{\bf{5}}}$	${\boldsymbol{y}}=-{\bf{1.26}}{\times {\bf{10}}}^{-{\bf{7}}}{\boldsymbol{x}}+{\bf{0.00101}}$

FA = fractional anisotropy; MD = mean diffusivity; FW = extracellular free water; NDI = neurite density index; ODI = orientation dispersion index; $\left|{{\mathrm{G}}}^{* }\right|$ = shear modulus. Bold font highlights significant *p*-values. Due to the low signal-to-noise ratio (SNR), grey matter for MWF data was excluded.

**Table 3.** Pearson’s correlation coefficients ($R$), *p*-values, and linear fit equations (white matter).

*x*	*y*	$R$	*p*-value	Linear fit equation
	$\left|{G}^{* }\right|$	**−0.61**	**0.0005**	${\boldsymbol{y}}=-{\bf{10.7}}{\boldsymbol{x}}+{\bf{2.95}}{\times {\bf{10}}}^{{\bf{3}}}$
	ODI	0.28	0.15	$y=0.000302{x}+\,0.339$
	NDI	0.29	0.13	$y=0.0004x+\,0.538$
Age	FA	**−0.39**	**0.047**	${\boldsymbol{y}}=-{\bf{0.000582}}{\boldsymbol{x}}+{\bf{0.351}}$
	FW	**0.49**	**0.012**	${\boldsymbol{y}}={\bf{0.00108}}{\boldsymbol{x}}+{\bf{0.121}}$
	MD	0.27	0.18	$y=4.49{\times 10}^{-7}{x}+\,0.000624$
	MWF	**0.43**	**0.028**	${\boldsymbol{y}}={\bf{0.000369}}{\boldsymbol{x}}+{\bf{0.0652}}$

	ODI	−0.37	0.051	$y=-2.33{\times 10}^{-5}x+\,0.411$
	NDI	−0.11	0.56	$y=-8.99{\times 10\,}^{-6}{x}+\,0.582$
$\left|{{\boldsymbol{G}}}^{* }\right|$	FA	0.38	0.055	$y=3.15{\times 10}^{-5}x+\,0.244$
	FW	−0.36	0.07	$y=-4.48{\times 10}^{-5}{x}+\,0.288$
	MD	−0.22	0.29	$y=-1.99{\times 10}^{-8}{x}+\,0.000696$
	MWF	−0.083	0.69	$y=-3.97{\times 10}^{-6}{x}+\,0.0962$

FA = fractional anisotropy; MD = mean diffusivity; FW = extracellular free water; NDI = neurite density index; ODI = orientation dispersion index; $\left|{{\mathrm{G}}}^{* }\right|$ = shear modulus; MWF = myelin water fraction. Bold font highlights significant *p*-values.

### 4.2. Correlations between shear modulus and other parameters

**Figure 4.**
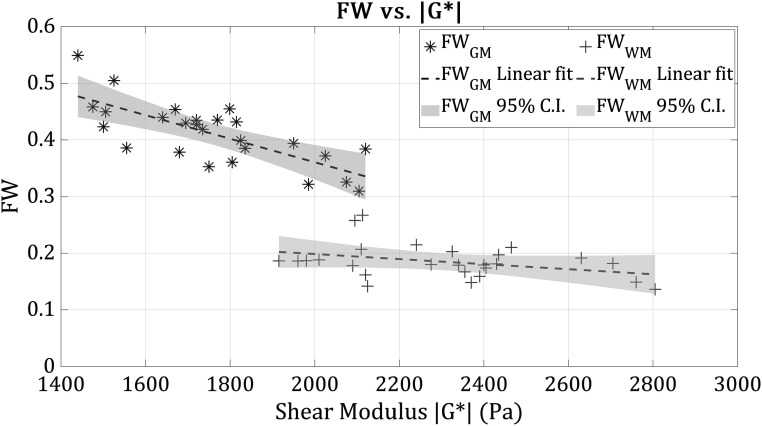
Free water (FW) measurement versus shear modulus across all subjects, without consideration of age, subjects’ shear modulus calculated from the average of reverberant + MDEV estimators, for grey (GM, in blue) and white (WM, in red) matter.

Here we seek the strongest link between shear modulus and the other measured MRI metrics, regardless of age. Among the measured parameters in this study, free water measures within the range of 0.1–0.6 across the subjects, produced the highest correlation with shear modulus, as shown in figure 4. Other parameters as a function of shear modulus are plotted in the appendix.

### 4.3. Models of shear modulus as a function of water content

**Figure 5.**
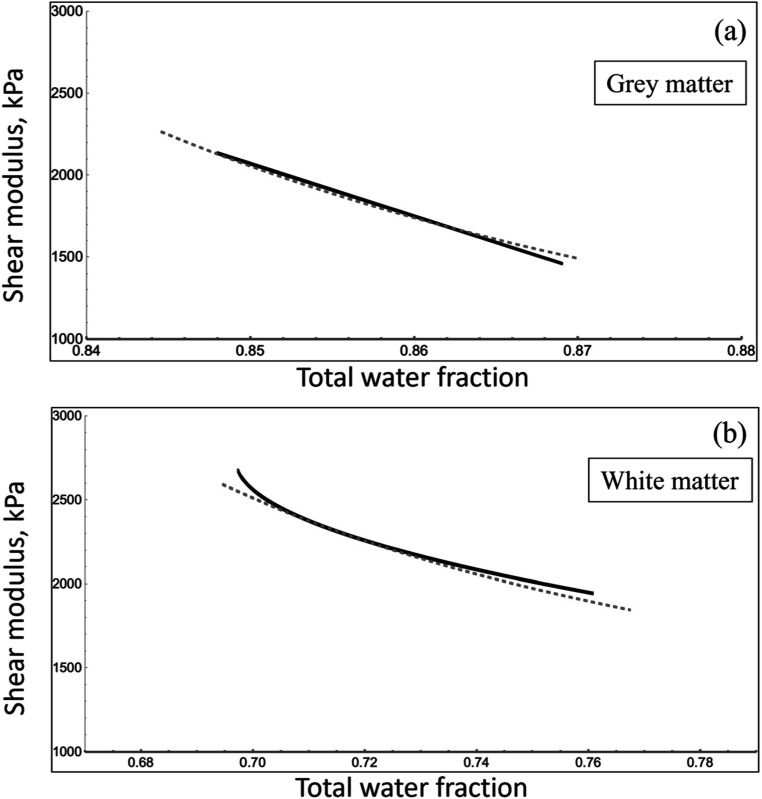
Shear modulus versus the total water percent weight, experimental and theoretical for grey matter (a) and white matter (b). We note that the range of the two plots are different, there is a higher total water fraction for grey matter. The solid (black) line is the trend line from measured populations. Age is a parametric (hidden) variable in this plot, but generally trends from left to right, meaning that older brains tend to have more water content and are softer. The dotted line is the theoretical curve fit using our MFM, and with a perturbation $1/{\chi }^{n}$ linking changes in shear modulus to changes exclusively within the extracellular water content around a central reference point, taken as approximately 0.85 and 0.70 total water weight fraction (grey and white matter, respectively) from adult middle age using the data of Svennerholm *et al* (1994). Importantly, the grey matter varies as $1/{\chi }^{6}$ but the white matter as $1/{\chi }^{2},$ indicating distinctly different mechanisms at work. Note that the scales on the two graphs cover different ranges.

In this section, we examine the results of the biphasic rheological model of the brain that was summarized in section 2 (Theory) and simplified to equation (2), against the trendlines for our study and that of Svennerholm *et al* (1994). We assume, *arguendo*, that our trendline for shear modulus versus age **(**figure 3 **)** and Svennerholm’s trendlines for dry/wet weight versus age (derived from figure 2(a)) provide jointly valid averages as a function of age. In that case, age between 20 and 80 years can be treated as a parametric variable, enabling a direct computation of shear modulus as a function of water fraction, and this inter-relationship is shown in solid (black) lines in figure 5(a) for grey matter and figure 5(b) for white matter. In theory, any small change in $\chi $ and $\unicode{x02206}W$ around a reference state can be modeled for first approximation by equation (2), and we assume the changes in the water fraction are assigned to the glymphatic system.

In particular, using Svennerholm’s values for water fraction in middle age (40–45 years) as our reference value, and assigning all the small fractional changes in water spaces, $\chi $ and water volume fraction $\unicode{x02206}W$ to the glymphatic system, assumed to be approximately 12% of the total water fraction at the reference conditions at middle age, our model for total water fraction in grey and white matter are:

\begin{eqnarray*}{W}_{{\mathrm{grey}}}=\displaystyle \frac{73.5+12{\chi }^{3}}{100}\end{eqnarray*}

and

\begin{eqnarray*}{W}_{{\mathrm{white}}}=\displaystyle \frac{58.5+12{\chi }^{3}}{100}\end{eqnarray*}

for grey and white matter water fractions, respectively. In this accounting, the water fractions at middle age (40–45 years) equal the trendline values from Svennerholm’s data, described in more detail in the appendix, equations (7) and (8). However, the small changes from this reference point are attributed to the extracellular spaces associated with the glymphatic system, assumed to hold a water fraction of 0.12 at the reference point (the other larger fluid volumes associated with blood, perivascular, and CSF spaces are not altered in this model). The most plausible agreements we found, in terms of dependence on $\chi $ to rational powers ($1/{\chi }^{n}$ where $n$ is an integer exponent) are shown in figure 5.

In these theoretical calculations (dashed lines), we note that the fit to grey matter is approximately proportional to $1/{\chi }^{6}$ or $1/{{\mathrm{\Delta }}W}^{2},$ whereas the fit to white matter is a lesser power law $1/{\chi }^{2}.$ This indicates that separate mechanisms are responsible for the trendlines in grey and white matter, and these will be detailed in the next section.

## 5. Discussion

### 5.1. The biophysics of trendlines

**Figure 6.**
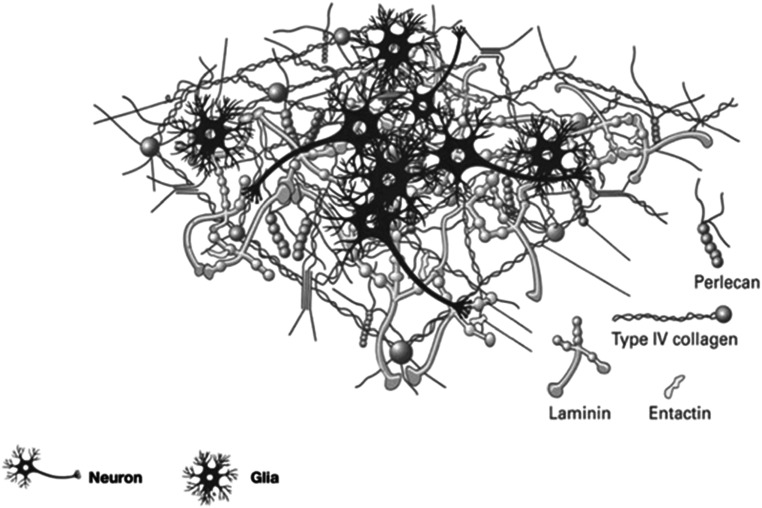
The basal lamina network is depicted schematically. Both laminin and collagen IV form a network resembling a sheet, along with other compounds forming an extracellular matrix that also permits fluid flow. In our rheological model, the aging brain is associated with a progressive loss of the structural components of the network, with free water increasing and shear modulus and ‘stiffness’ decreasing (Cieśluk *et al* 2022).

The major finding of this study is that within our framework, the softening of the aging brain and elastography measures can be plausibly tied to the progressive loss of proteoglycans, phospholipids, cholesterol, and other extracellular (and intracellular) components that are depicted in figure 6, with a corresponding increase in the fraction of water. This has the effect of softening the tissue, at least in terms of its response to shear waves within the frequency range commonly associated with elastography. The annual decrease in shear modulus we found, on the order of −10 Pa per year, is within the range of other MRE aging reports summarized by Hiscox *et al* (2021). However, we find the overall elastography trendline takes different mathematical form in grey matter versus white matter. Our model finds a plausible match of theory to trendline data by assuming that the major changes in water fraction are associated with the glymphatic system, where the extracellular fluid channels account for somewhere between 10% and 20% of the total fluid content of the brain (Rasmussen *et al* 2022). Tightly correlated with increases and decreases in this compartment over decades of age is the change in the shear modulus of the parenchymal matrix. The net result is that the shear modulus varies inversely as water fraction squared $1/{{\mathrm{\Delta }}W}^{2}$ in grey matter, but only as $1/{{\mathrm{\Delta }}W}^{\frac{2}{3}}$ in white matter. The grey matter results are consistent with trends found in some isotropic biomaterials (Nguyen *et al* 2014), whereas the white matter results are consistent with some anisotropic composite formulas which may model the white matter’s long axonal tracks. Specifically, the square root of volume fraction is a key parameter in elastic models of cylindrical inclusions. For example, the Chamis model of composite material with aligned cylinders (Chamis 1984) has been described as the ‘most widely used and trusted model’ (Younes *et al* 2012) and this model derives a shear modulus that varies as the square root of the volume fraction of the cylindrical inclusions. This trend, plus the fluid changes associated with demyelination, are plausibly responsible for the observed dependence on changes in shear modulus with age.

As a final comparison, it should be noted that general composite models, for example those outlined by Christensen (1969) and Lakes (1999), are not able to capture the relationship between $G$ and $W$ shown in figure 5. This inability has previously been depicted in figure A.1 of Ge *et al* (2023) for the aging murine brain, and strongly implies that more specific models, such as the framework developed herein, are required to capture the behavior of the aging brain.

### 5.2. Corresponding MR measures

Other MRI parameters measured in this study also seem consistent with earlier reported trends with aging (Motovylyak *et al* 2022). For grey matter $G,$ the strongest correlations we found included FW, FA, MD, and NDI, plausibly related to the trendlines shown in figures 1 and 2. However, for white matter, the link between extracellular free water and shear modulus is weak and not significant, as depicted in figure 4. The oriented axonal nature of white matter suggests a stronger link with measures such as fractional anisotropy but only weakly with myelin water fraction, a measure of water trapped in the myelin sheath. The strongest correlation we found within our set of measurements was the inverse relationship between free water and shear modulus as depicted in figure 4. This negative correlation is most pronounced in the grey matter where greater amounts of extracellular matrix (or glymphatic) free water are associated with softer grey matter, which is consistent with our earlier results both theoretical and experimental (Ge *et al* 2022, 2023) as detailed in the following section. Moreover, our results suggest a positive relationship between NDI and MD with age and stiffness in grey matter, while FA exhibits a negative correlation with stiffness and age in white matter. Conversely, ODI did not display any significant correlation. The increase in MD in grey matter with stiffness and age is expected due to increased isotropic diffusivity resulting from age-related edema or neuroinflammation, leading to increased extracellular free water. The decrease in FA with stiffness and age in grey matter is anticipated, while the elevated NDI in grey matter presents a contradiction, warranting further investigation with larger sample sizes. These age-related findings are in line with several prior studies (Kodiweera *et al* 2016), though inconsistencies across the literature may stem from variations in the age ranges utilized in these investigations (Billiet *et al* 2015, Kodiweera *et al* 2016, Merluzzi *et al* 2016, Slater *et al* 2019).

### 5.3. Of mice and men

There is a profound difference in trendline between the aging effects we found in mouse studies (Ge *et al* 2022, 2023) and this human study. Earlier experiments found that the mouse brain cortical grey matter stiffens and becomes drier (lower $W$) with age, whereas the human brain softens and has a higher water fraction $W$ as a function of age. However, at a deeper level of analysis, both species exhibit an inverse relation between grey matter shear modulus $G$ and ${{\mathrm{\Delta }}W}^{2}.$ Other studies on the younger developing mouse brain demonstrated a shift in properties towards more solid–elastic behavior during adolescent maturity (Guo *et al* 2019), whereas demyelination is associated with decreased stiffness (Schregel *et al* 2012). Any species differences must be investigated further, but our working hypothesis is that the noted opposite trends as a function of age are tied to the relatively short lifespan of mice, measured on the order of 30 months. Additionally, there is a reported thickening of the basement membranes of the brain with age over the short murine lifespan. The basement membranes comprise a thin but widely distributed extracellular matrix within the blood–brain barrier and have been found to double in thickness with age in rodent brains. The membranes thicken in response to mechanical stresses and can develop altered lipid, laminin, fibronectin, and other proteoglycan components (Ceafalan *et al* 2019, Reed *et al* 2019). In marked comparison, the long-term dysregulation and inflammation of the brain in humans along with the progressive loss of solid weight fraction leads to the accelerating increase in water fraction towards the later decades of human life. Additional effects in the aging human brain that can influence viscoelastic measures include *ß*-amyloid burden, white matter microbleeds, enlarged perivascular spaces, and gradual decrease in neuronal density (Hiscox *et al* 2021). Overall, our MFM biphasic model with the approximation for small changes in fluid spaces (and fluid volume) around a reference point, is able to capture the trendlines for both mice and human aging brains within a common underlying mathematical framework.

### 5.4. Other factors

We note that the results and model in this study pertain to long-term changes extending over decades. This is very different from the short-term sleep/wake cycles that have been shown to produce changes in stiffness of cortical grey matter of mice (Ge *et al* 2022). It remains to be seen if human sleep/wake cycles do produce short-term cycling of elastography measures, and this is a topic for future work. The reported $G$ values in the results sections are the average of MDEV and RSWE estimators. We found that the correlation $r$ between these two estimators was greater than 0.9 across the entire population. RSWE values of $G$ were, on the average, approximately 10%–15% higher than MDEV estimates for the same subject, and the detailed reasons for this will require further research.

Limitations of this study include the limited numbers available (*N* = 28) and the lack of an independent gold-standard measure of water fraction within the extracellular matrix of grey and white matter. Partial volume effects are expected to be minimal for the healthy cohort utilized in this study. Additionally, while efforts were made to mitigate partial volume effects for more accurate estimation of MRI metrics, specifically extracellular free water, it remains possible that unavoidable partial volume effects may occur due to fluid in the subarachnoid space. We utilized the trendlines from Svennerholm’s landmark study as a population average, but individual variations would be desirable for fine-tuning of the model and for understanding the role of co-factors. Future work should explore models that include the particular roles of the DTI and NODDI parameters including FW, FA, MD, and myelin water imaging parameters such as MWF which have varying degrees of correlation with elastography $G$ but each pertain to distinct microstructural factors. Also, measures of the vascular and perivascular spaces should be included in future models. Finally, the larger goal relegated for future work is the study of how disease states and injuries change the elastography measures and if the model presented herein can capture these changes with or without significant additional terms.

## 6. Conclusion

The long-term trend towards softening in the aging human brain has now been modeled as a tight coupling between the progressive loss of solid fraction with the concomitant gain in water fraction, resulting in a softening of the brain especially approaching the 7th decade and beyond. In some respects, these trends resemble the game of *Jenga* (the game of incremental removal of building blocks while avoiding collapse), played over decades on the extracellular or basement membrane level, with corresponding decrease in shear modulus, a softening effect. Elastography thereby produces a unique macroscopic view of a microscopic structural change that has important implications for function and dysfunction, for example the efficient functioning and fluid flow within the glymphatic system (Rasmussen *et al* 2022). The shear modulus under our model framework is also consistent with trends found by other MRI measures (free water, fractional anisotropy, and others), however elastography provides a direct link to the physical stress–strain response of the parenchyma and its role in fluid transport and response to mechanical forces. The trendline for any individual’s elastography results can provide an insightful biomarker of the accumulation of fluids at the expense of the parenchymal matrix solid fraction that peaks in healthy young adults.

## Figures and Tables

**Figure A.1.**
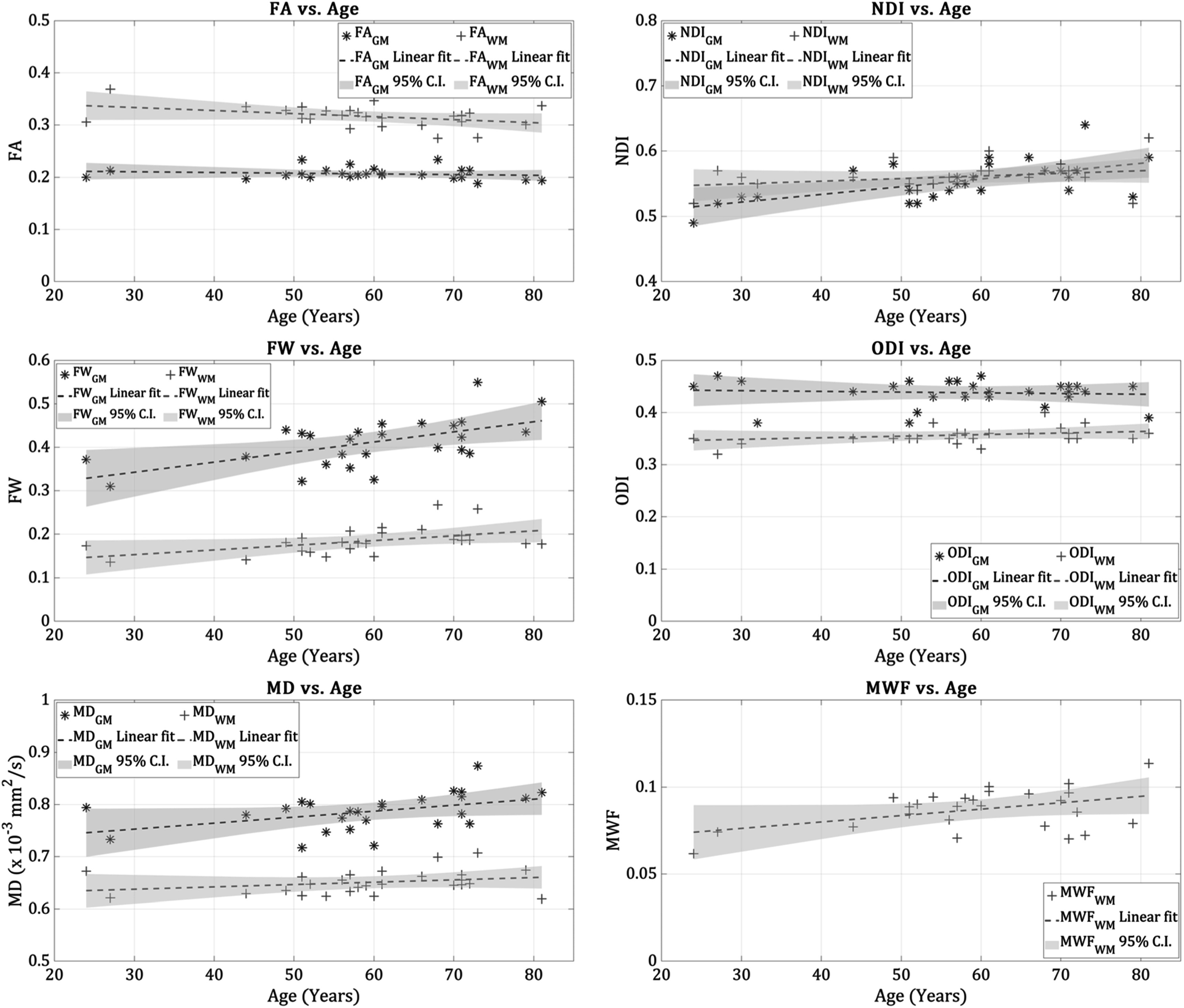
Scatter plots depicting the correlations between MRI metrics and age in both grey and white matter. Dashed lines indicate linear fits.

**Figure A.2.**
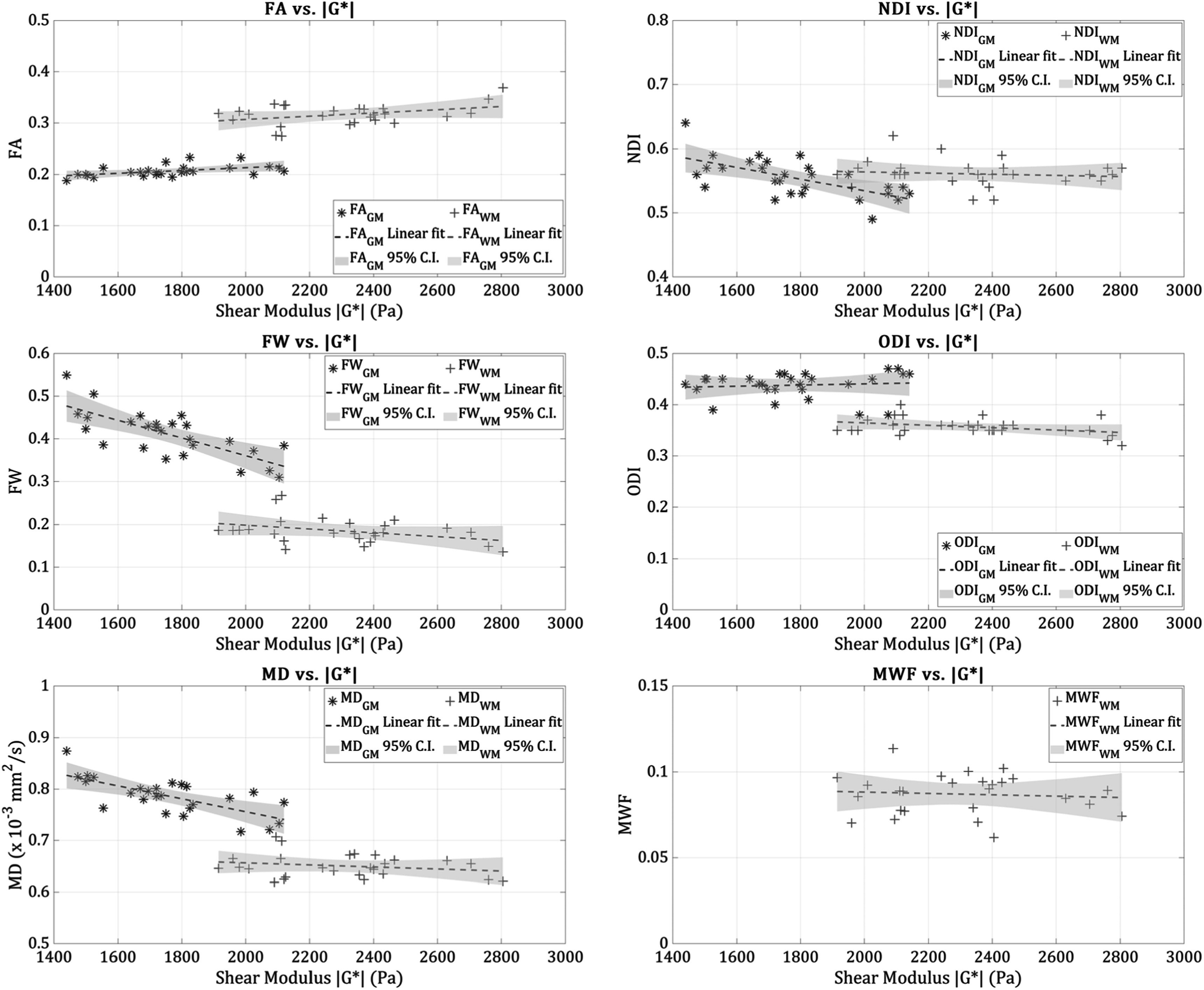
Scatter plots depicting the correlations between MRI metrics and shear modulus in both grey and white matter. Dashed lines indicate linear fits. GM: grey matter; WM: white matter.

## Data Availability

All data that support the findings of this study are included within the article (and any supplementary information files).

## Appendix

For completion we include the measured parameters from MRI as a function of age (figure A.1) and then as a function of estimated shear modulus $G$ (figure A.2), with linear correlation fits for both grey matter and white matter. Those linear fit equations can be found in tables 2 and 3.

Next, we include the equations derived from Svennerholm *et al* (1994), where we assume that the water fraction $W$ is given as (1—the solid fraction). The solid fraction is presented in graphical form and in tabular form by Svennerholm along with their first and second order fits (with some errors in decimal point placement). From these, we utilize the following functions in our models:

\begin{eqnarray*}\begin{array}{c}{\mathrm{white}}\,{\mathrm{matter}}\,W=\left[1-\left(0.303-0.000013{\left({\mathrm{age}}-20\right)}^{2}\right)\right]\\ {\mathrm{where}}\,20\,< \,{\mathrm{age}}\,< \,90\,{\mathrm{years}},\end{array}\end{eqnarray*}

and

\begin{eqnarray*}\begin{array}{c}{\mathrm{grey}}\,{\mathrm{matter}}\,W=\left[1-\left(0.152-0.0003\left({\mathrm{age}}-20\right)\right)\right]\\ {\mathrm{where}}\,20\,< \,{\mathrm{age}}\,< \,90\,{\mathrm{years}}.\end{array}\end{eqnarray*}

We note that, consistent with their plotted data and trendlines, the trendline for white matter is essentially second order, accelerating in later years, whereas the trendline for grey matter is well-represented by a simple first order curve. These equations, along with equation (4), provide the means for producing the parametric plots of $G$ versus $W,$ shown in figure 5, representing the population trendlines.
